# Essential Oils and Isolated Terpenes in Nanosystems Designed for Topical Administration: A Review

**DOI:** 10.3390/biom9040138

**Published:** 2019-04-05

**Authors:** Sheila P. de Matos, Helder F. Teixeira, Ádley A. N. de Lima, Valdir F. Veiga-Junior, Letícia S. Koester

**Affiliations:** 1 Programa de Pós-Graduação em Ciências Farmacêuticas, Faculdade de Farmácia, Universidade Federal do Rio Grande do Sul, Av. Ipiranga, 2752, Porto Alegre 90610-000, Brazil; sheilaporto@outlook.com (S.P.d.M.); helder.teixeira@ufrgs.br (H.F.T.); 2 Programa de Pós-Graduação em Ciências Farmacêuticas, Departamento de Farmácia, Universidade Federal do Rio Grande do Norte, Av. General Cordeiro de Farias, s/n, Petrópolis, Natal 59012-570, Brazil; adleyantonini@yahoo.com.br; 3 Departamento de Engenharia Química, Instituto Militar de Engenharia, Praça Gen. Tibúrcio, 80, Praia Vermelha, Urca, Rio de Janeiro 22290-270, Brazil; valdir.veiga@gmail.com

**Keywords:** essential oils, terpenes, nanotechnology, cyclodextrins, topical drug administration

## Abstract

Essential oils are natural products with a complex composition. Terpenes are the most common class of chemical compounds present in essential oils. Terpenes and the essential oils containing them are widely used and investigated by their pharmacological properties and permeation-enhancing ability. However, many terpenes and essential oils are sensitive to environmental conditions, undergoing volatilization and chemical degradation. In order to overcome the chemical instability of some isolated terpenes and essential oils, the encapsulation of these compounds in nanostructured systems (polymeric, lipidic, or molecular complexes) has been employed. In addition, nanoencapsulation can be of interest for pharmaceutical applications due to its capacity to improve the bioavailability and allow the controlled release of drugs. Topical drug administration is a convenient and non-invasive administration route for both local and systemic drug delivery. The present review focuses on describing the current status of research concerning nanostructured delivery systems containing isolated terpenes and/or essential oils designed for topical administration and on discussing the use of terpenes and essential oils either for their biological activities or as permeation enhancers in pharmaceutic formulations.

## 1. Introduction

Essential oils (EO) are natural products extracted by hydrodistillation from plant materials, composed by small, volatile, and fairly hydrophobic molecules [1,2,3]. The functions of EO in plant organisms seem to be related to environmental interactions and protection of the plant against predators and pathogens [1,4,5]. In the industry, essential oils are materials of great interest with a wide range of possible applications in the nutritional, agricultural, cosmetic, and pharmaceutical fields [6,7], due to their broad spectra of biological activities such as antimicrobial, repellent, analgesic, anti-inflammatory activity, and many others [1,2]. Essential oils can be extracted from plant matrices using different techniques classified as classical/conventional (that use water distillation by heat as a means to extract the whole volatile material) and innovative/advanced (which focus on the improvement of selectivity in extraction efficiency by reducing extraction time, use of energy, solvent, and CO_2_ emissions) [1]. 

Terpenes and terpenoids (the oxygenated derivatives of terpenes [2]) are the chemical compounds representing the majority of molecules in essential oil composition [8]. This class of molecules is characterized by the combination of isoprene units (C_5_H_8_). Terpenes can be classified according to the number of isoprene units in their structure (e.g., hemiterpenes are formed by one isoprene unit, monoterpenes are formed by two isoprene units, sesquiterpenes by three isoprene units, and diterpene formed by four units) [2,8]. Smaller terpenes, up to three isoprene units, are highly volatile, and the volatility decreases with an increased number of isoprene units [9]. Many biological activities of terpenes and terpenoids are described in the literature, being this class of molecules a valuable source of therapeutic agents with pharmaceutical applications, such as anti-inflammatory [10], wound healing [11], antineoplastic applications [12,13,14]. In addition, some compounds of the class are widely investigated and reported as penetration enhancers for percutaneous drug delivery, being used as excipients in the preparation of nanostructured systems [7,8,15]. Smaller terpenes are usually thermolabile and susceptible to volatilization and degradation, mainly by oxidation and isomerization [6,16]. 

The topical administration of drugs consists in the localized administration of formulations to an organism, comprising dermal and mucosal administrations (e.g., ocular, vaginal, nasal, and rectal routes). Among those, the most widely employed is the cutaneous route, which may be attributed to the easiness of access compared to other topic routes [17]. When applied to the skin, a drug delivery system can be considered dermal (when the targeting site of the drug is the skin) or transdermal (when the drug needs to pass through skin layers in order to reach its target); for mucosal tissue administration, delivery can be mucosal and transmucosal [18]. Many issues concerning these delivery routes are described in the literature, such as the resistance to diffusion through the skin and mucosae when aiming towards transdermal or transmucosal delivery and the reduced contact of the formulation with mucosal tissues due to mucus and, thus, reduced drug bioavailability [19,20].

Encapsulation of essential oils in micro or nanometric systems is an interesting strategy to provide better stability to the volatile compounds and protect them against environmental factors that may cause chemical degradation [2,6]. In addition, the encapsulation in nanometric systems may improve the bioavailability and bioefficacy of formulations as a result of cellular absorption and provide controlled release of bioactive compounds [3,21]. Special nanostructured systems have been designed intending topical administration as approaches to overcome the drawbacks inherent to these administration routes, such as mucoadhesive systems that prolong the contact with mucosae and systems that favor the passage of the drug trough skin or mucosal tissue layers [20,22]. Molecular encapsulation through complexation with cyclodextrins is another strategy employed to improve the stability of essential oil components, avoid volatilization (and, consequently, prolonging contact,) and enhance permeation of the bioactive molecules [23]. 

In recent decades, the interest in nanotechnological approaches and use of natural products have raised great attention in the search and development of drug delivery systems. The present review intends to assess the panorama of research using nanostructured delivery systems containing essential oils and/or their isolated terpenes/terpenoids as bioactive compounds and/or excipients for topical administration routes. 

## 2. Literature Survey

A literature survey was carried out in three different databases: Embase, Scopus, and Pubmed. Initially, all results found until 31 December 2018 were considered, without limiting the search period before this date. The search terms used were a combination of words related to nanostructured systems “AND” terpenes/essential oils “AND” topical administration route. All classes of terpenes were included, not only low-volatile terpenes, as well as all topical administration routes, which comprise cutaneous and mucosal administration. Database search lines can be found in Supplementary Materials, Table S1. 

**Figure 1:**
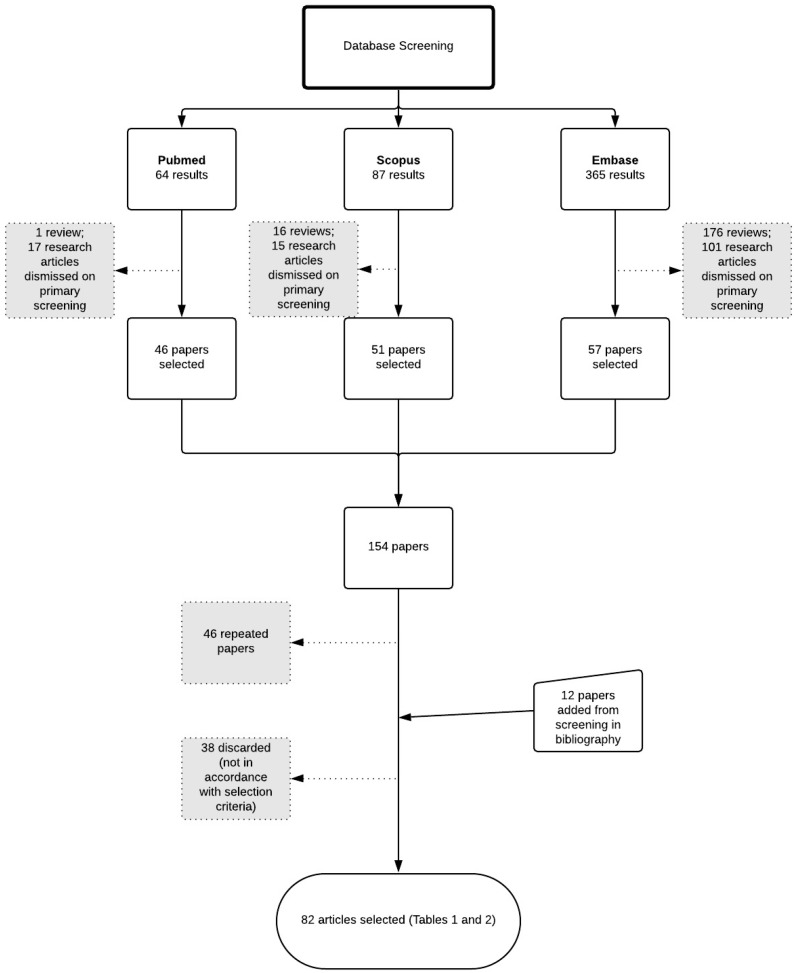
Flowchart representing the steps of literature survey and selection of research papers.

The number of results obtained using Scopus were 87, those from Embase were 365, and those from PubMed were 64. Among those results, only research articles in English were considered for data screening. Also, duplicates were disregarded. In addition, reference lists of papers were screened to detect research papers which did not appear in the database research but might fulfill the acceptance criteria (Figure 1). Afterwards, the papers were screened and selected if meeting the following acceptance criteria:

- Original research data.
- Use of essential oil containing terpenes and/or isolated terpenes in nanostructured systems or cyclodextrins.
- Formulations for topical administration.

**Figure 2:**
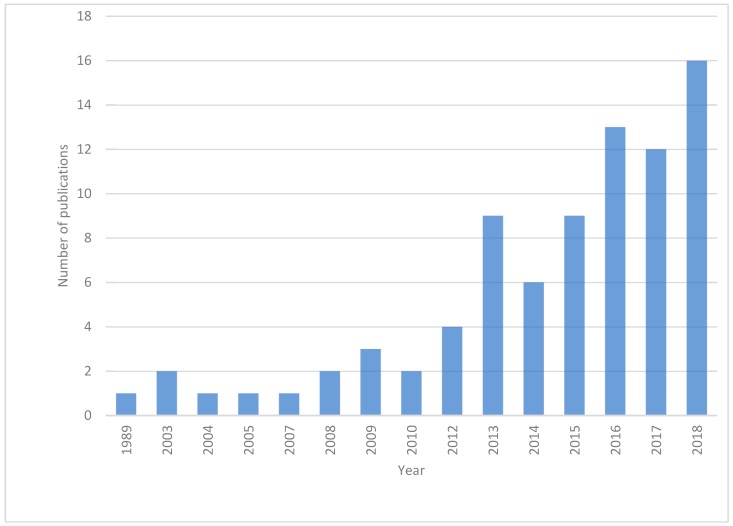
Annual distribution of publications.

Finally, 82 papers were selected for further data extraction. Studies were sorted by year of publication in order to visualize the evolution of research in this field over the years (Figure 2). The first report dated from 1989 and was by Saettone et al. [24], who studied the complexation of the diterpene forskolin with cyclodextrin for ocular administration. The number of publications has gradually grown over the years, indicating an increasing interest in the topic.

Data concerning the essential oil or terpene used, encapsulation system, nanosystem preparation technique, administration route of formulation, and use of EO/terpene as bioactive compounds or excipients were extracted from the selected papers and compiled in Table 1 and Table 2 in order to ease the access to the extracted data, which are further discussed in the following sections of this review.

## 3. Discussion

### 3.1. Topical Administration Routes

**Figure 3:**
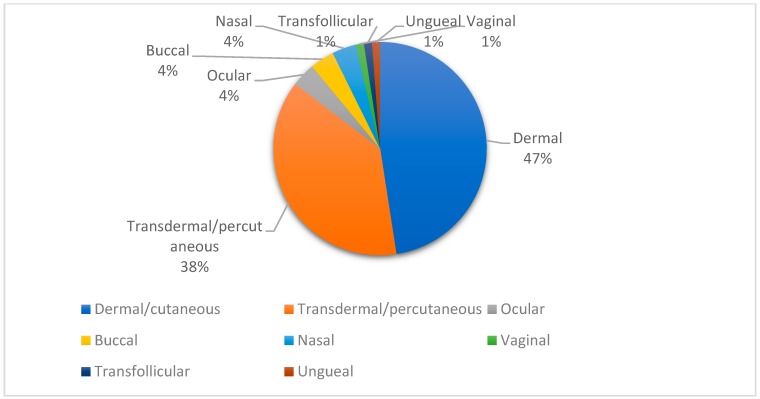
Topical administration routes of nanoencapsulated essential oils (EO) containing terpenes and isolated terpenes.

Topical drug administration can be described as the localized application of a pharmaceutical dosage form and comprises both dermal and mucosal administration [19,20]. In Figure 3, the different sites of administration for nanosystems containing isolated terpenes and/or EO are shown. It can be noticed that among the study formulations containing nanoencapsulated EO and/or terpenes, dermal and transdermal administration represent nearly 90% of the administration routes, which can be attributed to the non-invasiveness, convenience, and painless qualities of this type of administration.

Dermal administration of nanostructured systems containing terpenes and EO is, in general, mainly used as a treatment for conditions localized on the skin surface, such as for wound healing, and to vehiculate anti-inflammatory, antimicrobial, and repellent agents. On the other hand, transdermal delivery is employed in cases where the bioactive compound needs to reach deeper layers of the skin or even the systemic circulation, such as for anesthetic, antihypertensive, and antidiabetic drugs (Table 1 and Table 2).

Mucosal administration (ocular, nasal, oral, vaginal) still represents a challenging route due to the presence of mucus, lacrimal fluids, and saliva, which can impair the bioavailability of the bioactive compound [19]. However, it is possible to foresee the development of mucoadhesive formulations as means to overcome these limitations.

### 3.2. Role of Essential Oil and/or Terpenes in the Formulations

**Figure 4:**
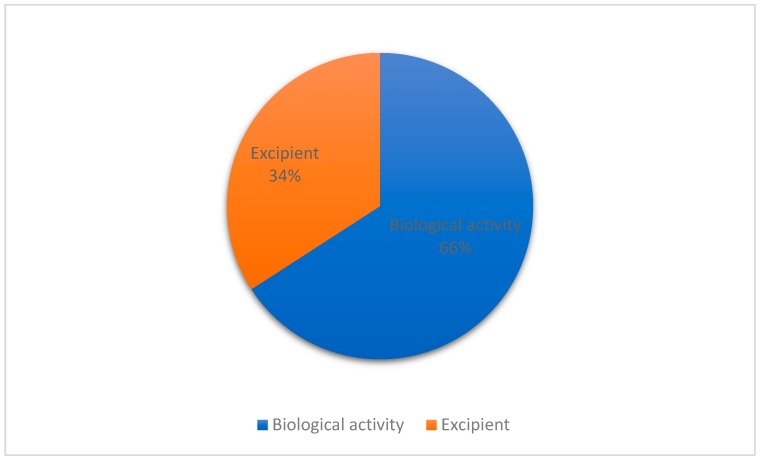
Application of EO/terpenes as excipients or bioactive ingredients in formulations.

In order to understand the applications of EO containing terpenes and/or isolated terpenes encapsulated in nanosystems intended for topical administration, the studies were divided between those in which EO/terpenes were used as excipient and those in which they were used as bioactive compounds. Of 82 studies, 56 employed terpenes/EO as bioactive ingredients in formulations, whereas 29 reported terpenes/EO as excipients in formulations (Figure 4). Zhang et al. [38] reported both bioactive (anti-inflammatory) and penetration-enhancing activities of the terpene Paeoniflorin encapsulated in glycerosomes. Also, Castangia et al. [90] described the preparation of permeation-enhancing vehicles containing *Santolina insularis* EO as an antimicrobial and permeation enhancer for percutaneous drug delivery. 

Many biological and pharmacological activities of EO and terpenes are well described in the literature, and the interest in their use as bioactive ingredients in pharmaceutical formulations has been rising recently, alongside with the seek for natural products as alternatives for drug development [1,5].

In general, the use of terpenes as excipients aims to increase the permeation of the active compound through skin layers, since terpenes are extensively cited in the literature as permeation enhancers [7,15,102]. The application of terpenes as excipients is further discussed in Section 3.2.2.

#### 3.2.1. Nanostructured Systems Used in the Encapsulation of Terpenes and Essential Oils as Bioactive Ingredients

The therapeutic potential of terpenes and EO containing terpenes is well known. Biological activities such as bactericidal, fungicidal, antioxidant, virucidal, and antineoplastic activities are well recognized [3,5,103]. There is a number of good reviews in the literature that list the variety of nanostructured systems and preparation techniques employed in the encapsulation of EO in nanostructures systems [1,2,104], but none focused on terpenes. Among the studies listed in Table 1 and Table 2, the most widely investigated biological activities upon nanoencapsulation of essential oils and isolated terpenes were the anti-inflammatory activity of essential oils and oil resins containing terpenes, such as plai oil [93], *Copaifera multijuga* oil [89,97], *Nigella sativa* EO [78], and of the isolated terpenes triptolide [41,65,71,73], sericoside [72], thymol [10] and oleanoic and ursolic acids [53], as well as the wound healing properties of *Syzygium aromaticum* EO [83], *Melaleuca alternifolia* EO [91], triterpenes from *Centella asiatica* extract [85], madecassoside [105], asiaticoside [48], astragaloside IV [59], and hyperforin [11]. Also, the nanoencapsulation of the well-established taxane antineoplastic drugs paclitaxel [14] and docetaxel [13,35] were studied for topical administration.

The systems can be divided according to their composition of polymers and lipids [21]. Polymer-based systems can be constituted by natural, synthetic, and semisynthetic polymers and comprise systems such as nanocapsules (NC), nanoparticles (NP), nanofibers (NF), and nanogels (NG). On the other hand, the lipid-based systems, namely, nanoemulsions (NE), liposomes (LS), solid lipid nanoparticles (SLN), nanostructured lipid carriers (NLC), and vesicular systems (VS) which comprehend ethosomes, phytosomes, niosomes, glycerosomes, and invasomes (IV) [106], are formed by lipids [104]. In addition, molecular complexation of terpenes with cyclodextrins is reported in the literature as a strategy for essential oil and natural products nanoencapsulation [2,107]. 

Encapsulation in nanometric systems is widely described in the literature as an approach to overcome many disadvantages concerning essential oils by providing protection against environmental factors that can cause chemical degradation and avoiding volatilization of EO components. In addition, nanoencapsulation can improve the bioavailability, allow controlled drug release, and enable the passage of the bioactive compounds through biological barriers [21]. Patel et al. [62] compared the cutaneous permeation of the sesquiterpene Hurpezine A from transdermal gels after encapsulation in three different systems, i.e., microemulsion (ME), SLN, and NLC, and the in vivo activity in an experimental mice model of induced amnesia. The ME formulation presented the highest cumulative permeated amounts, followed by NLC and SLN. The in vivo experiments presented no significant difference between the three formulations, but positive outcomes when compared to the negative control. In other study of comparison between different nanostructured systems, Flores et al. [96] investigated nanocapsule suspensions and nanoemulsions containing tea tree oil against in in vitro onychomycosis models. Although both nanometric systems presented better antifungal activity compared to the coarse emulsion containing tea tree oil, the nanocapsule formulation was more effective against the microorganism. This may be attributed to the polymer barrier that prevents the volatilization of bioactive compounds as well as the capacity of the aqueous suspension to hydrate the tissue and therefore enhance the penetration of nanostructures. 

Also, the complexation with cyclodextrins can be an interesting alternative, since it is reported to provide protection against environmental factors, improve stability, avoid volatilization of EO components, improve oral bioavailability, and reduce mucosal irritation [23]. In addition, cyclodextrins are described as safe options to enhance the permeation of molecules through the skin and mucosae [2,15,108]. The complexation of essential oils and isolated terpenes with cyclodextrins has been reported in the literature and reviewed [23,109,110]; however, the majority of investigations concerning complexes between cyclodextrins and essential oils or isolated terpenes for pharmaceutical applications are limited to oral administration, with few studies focusing on topical administration, and no reviews found in the literature covering this subject specifically. Rode et al. [72] studied the influence of cyclodextrin complexation on the percutaneous penetration in pig skin of the triterpenoid sericoside from a *Terminalia sericea* extract. The formulation containing the sericoside from the extract complexed with γ-cyclodextrin increased the percutaneous penetration 2.6 times when compared to an ethanolic solution of sericoside.

**Figure 5:**
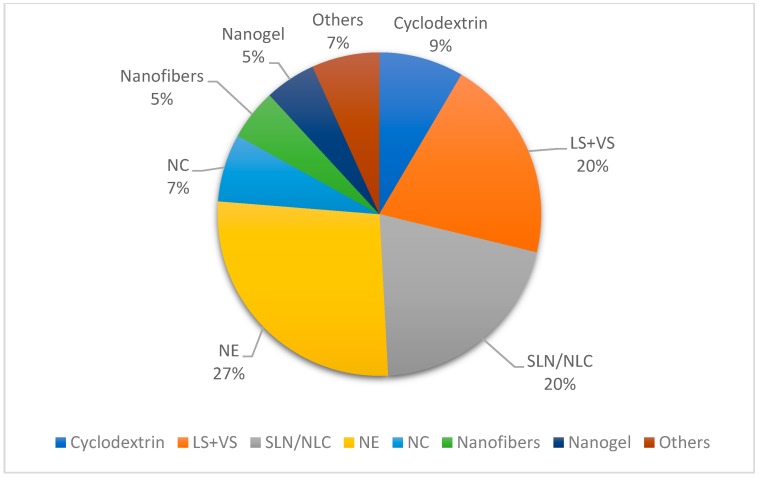
Nanostructured systems used in the encapsulation of essential oils containing terpenes and isolated terpenes as bioactive compounds for topical formulations. LS, liposomes, VS, vesicular systems, SLN, solid lipid nanoparticles, NLC, nanostructured lipid carriers, NE, nanoemulsions, NC, nanocapsules.

As illustrated in Figure 5, a variety of different nanostructured systems are used in the encapsulation of EO and terpenes. It can be noticed that lipid-based nanosystems are used in 67% of the formulations reported, which may be attributed to their low toxicity.

Although various preparation techniques of nanostructured systems are extensively described in the literature [1,2,104], it is important to pay attention to the preparation technique in terms on its influence on EO/terpene stability, because of their sensitivity to heat and pressure. In this context, the quantification of chemical markers of EO or terpenes is essential, especially in cases where they play a role as bioactive compounds, in order to ensure the integrity of EO/terpene content in the final formulation and, thus, the safety and efficacy of the final product [104].

#### 3.2.2. Terpenes as Excipients in Formulations for Topical Administration

Transdermal drug delivery represents a convenient route of administration for systemic action due to its non-invasive characteristic [102,111]. However, it is limited by the ability of the drug to permeate through the skin layers, especially the stratum corneum (SC). The use of penetration enhancers is a promising strategy to broaden the range of drugs eligible for transdermal delivery [112]. 

Permeation enhancers are used as excipients in formulations and are capable of facilitating the passage of a drug through the skin. Besides their biological activities, essential oils and isolated terpenes have been described as promising permeation enhancers for transdermal drug delivery. They can enhance the passage of drugs through the skin by interacting with stratum corneum lipids and modifying the solubility characteristics of these lipids, facilitating drug partitioning towards deeper layers of the skin [7,8,15,102]. Furthermore, terpenes are considered safer than other permeation enhancers from a toxicological point of view, with some terpenes being included in the list of Generally Recognized as Safe (GRAS) compounds from the US FDA (United States Food and Drug Administration) [8].

**Figure 6:**
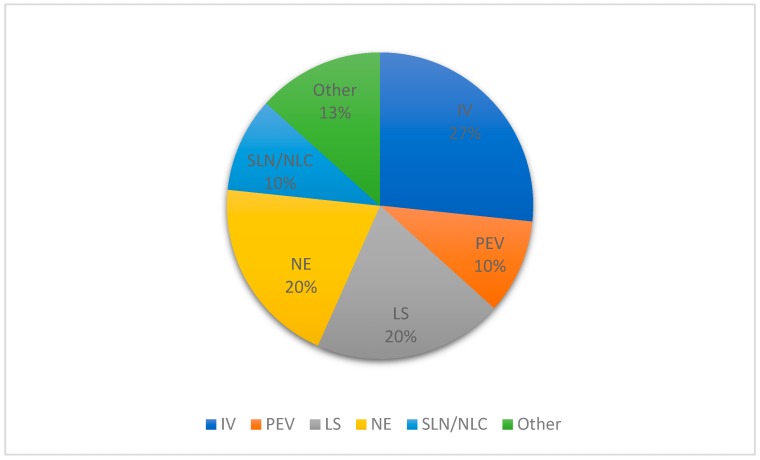
Nanostructured systems used in formulations containing essential oils and/or isolated terpenes excipients for topical administration. IV: invasomes, PEV: permeation-enhancing vehicles.

The nanostructured systems in which terpenes are used as excipients are illustrated in Figure 6. The majority of systems are based on vesicular structures (LS, I,V and permeation-enhancing vehicles (PEV)), with the invasomes representing 27% of the nanostructured systems reported, followed by liposomes and nanoemulsions (20%).

**Figure 7:**
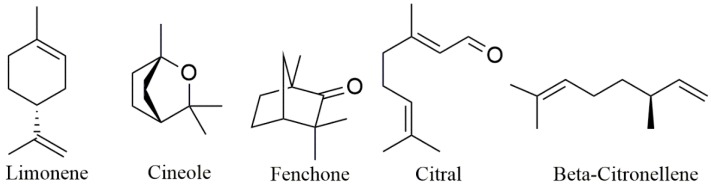
Monoterpenes used as permeation enhancers in nanostructured systems.

Invasomes (also found in the literature as invasosomes) are special drug delivery systems that contain terpenes and are the most recurrent among the formulations of nanosystems using terpenes as excipients (Figure 6). They are, by definition, vesicular systems consisting of terpenes, ethanol, and phospholipids as fundamental raw materials and present elasticity and deformability, which favors penetration across skin layers, and thus they work as penetration-enhancing vehicles [113]. According to the data presented in Table 1 and Table 2, the terpenes used in the formulation of invasomes are limonene, cineole, fenchone, citral, and beta-citronellene; all of them are monoterpenes or monoterpenoids (Figure 7). 

Kamran et al. [45] studied the preparation of invasomes using beta-citronellene as a permeation enhancer for transdermal delivery of the antihypertensive olmesartan and observed an increase in the bioavailability of olmesartan from invasomes in Wistar rats compared to the control. Incorporation of isradipine, also an antihypertensive agent, in invasomes containing beta-citronellene was investigated by Qadri et al. [42], who showed a decrease of 20% of blood pressure in rats. The permeation of rhodamine B invasomes through rat skin was evaluated by confocal laser spectroscopy, and fluorescence was detected at a depth of 171.18 mm in the skin. A series of studies focused on the encapsulation of temoporfin in invasomes, aiming to improve its penetration through the skin using several different proportions of cineole, citral, and d-limonene. The findings indicated that the optimal amount of terpenes in formulations should be 1% (v/v), and the formulation containing 1% of cineole alone led to higher permeation of the drug to deeper layers of the skin compared to mixtures of the three terpenes in varied proportions and limonene and citral alone [68,69,70]. El-Nabarawi et al. [25] prepared dapsone-loaded invasomes for acne treatment, containing different terpenes (limonene, cineole, citral, or fenchone) in varied concentrations in a full factorial design study and compared drug deposition in vivo of optimized formulations with dapsone solution, finding a 2.5-fold increase in dapsone deposition with the invasomes formulation.

### 3.3. Safety of Essential Oils and Terpenes in Topical Administration

One important concern is the safety of the use of essential oils and terpenes in topical formulations. Although a number of essential oils and terpenes are considered GRAS [8], few studies investigated the safety of these compounds in topical application. Lalko and Api [114] investigated the skin irritancy of essential oils and their isolated compounds, concluding that, in many cases, there is a potential induction of sensibilization upon exposure to these compounds. Some terpenes, as described in previous sections of this review, are used as permeation enhancers in drug delivery systems. They are considered non-toxic, nonirritant, and, therefore, a safe option for permeation enhancement [115].

### 3.4. Sustainability

In recent decades, the efforts to reduce the environmental impact of chemical compounds and develop green chemistry have been growing. Terpenes and essential oils comprise a renewable source of chemical compounds and have been already described in the literature as “greener” alternatives to pesticides [116] and solvents [117]. Concerning the preparation of nanostructured systems, essential oils and terpenes can be used as more sustainable additives, being able to substitute organic solvents in the preparation of nanocapsules [118] and in the synthesis of metallic nanoparticles [119]. Also, effort has been made for the development of essential oil extraction techniques with no or less solvent and energy use without quality loss [120]. When observing the 12 principles of Green Chemistry [121], it is possible to see that the use of terpenes and essential oils may have some positive effects, since, as aforementioned, they are renewable supplies and present low toxicity [8]. Furthermore, in the development of new formulations containing essential oils and terpenes, the 12 principles (prevention, atom economy, less hazardous chemical synthesis, designing safer chemicals, safer solvents and auxiliaries, design for energy efficient, use of renewable feedstocks, reduce derivatives, catalysis, design for degradation, real-time analysis for pollution prevention, and inherently safer chemistry for accident prevention) should be respected, since, besides reducing the environmental impact of chemicals, they can also lead to lower costs of production [122], which can balance out investments made in the development and implementation of sustainable alternatives.

## 4. Concluding Remarks

The interest in the development of nanostructured systems containing essential oils and terpenes designed for topical administration routes has been rising in recent years. The majority of the formulations employ EO and/or terpenes as active ingredients encapsulated in nanostructured systems as means to improve the physicochemical properties and/or achieve greater bioavailability and controlled release of drugs. On the other hand, a part of the studies also shows the growing interest in using terpenes as permeation-enhancer excipients in transdermal delivery systems, a promising strategy for efficient and noninvasive drug delivery to the skin deeper layers.

Terpenes and essential oils present a sustainable alternative as raw materials in the pharmaceutical field and thus a valuable source, giving the growing importance of “greener” chemistry. Many of them are considered GRAS; however, there is a lack of information concerning their safety in topical administration.

## Figures and Tables

**Table 1:** Publications found in the literature concerning the encapsulation of isolated terpenes in nanostructured systems intended for topical administration.

	Year	Terpene	System	Administration Route	Biological Activity	Terpene Role in the Nanosystem
[25]	2018	Limonene, cineole, fenchone, and citral	Invasomes	Cutaneous	Anti-acne	Excipient
[26]	2018	Ursolic acid	Solid lipid nanoparticles	Cutaneous	Antiarthritic	Bioactive
[27]	2018	Limonene	Nanovesicles	Transdermal	Antineoplastic	Excipient
[28]	2018	Paeoniflorin	Ethosomes	Transdermal	Antiarthritic	Bioactive
[29]	2018	Rebaudioside A	Ultra-small micelles	Ocular	NA	Excipient
[30]	2018	Farnesol	Nanoparticles	Oral	Antibiofilm	Bioactive
[31]	2018	Eucaliptol	Nanoemulsion	Transfollicular	NA	Excipient
[32]	2018	Menthol	Nanoparticles	Transdermal	Osteoporosis treatment	Excipient
[33]	2018	Tripterine	Phytosomes	Oral	Antineoplastic	Bioactive
[34]	2018	Ursolic acid and anethole	Liposomes	Nasal	Antineoplastic	Bioactive and excipient
[10]	2018	Thymol	Solid lipid nanoparticles	Cutaneous	Anti-inflammatory	Bioactive
[35]	2018	Docetaxel	Polymeric nanoparticles	Nasal	Antineoplastic	Bioactive
[36]	2018	Forskolin	Nanostructures lipid carriers	Transdermal	Photoprotector	Bioactive
[37]	2017	Citral and limonene	Transferosomes and liposomes	Transdermal	Antiarthritic	Excipient
[38]	2017	Paeoniflorin	Glycerosomes	Transdermal	Anti-inflammatory	Bioactive and Excipient
[39]	2017	Limonene	Transinvasomes	Transdermal	NA	Excipient
[40]	2017	Limonene	PEGylated liposomes	Transdermal	Alzheimer’s treatment	Excipient
[41]	2017	Triptolide	Nanoemulsion	Percutaneous	Anti-inflammatory and analgesic	Bioactive
[42]	2017	β-citronellene	Invasomes	Transdermal	Hypertension treatment	Excipient
[43]	2017	α-bisabolol	Nanocapsules	Ocular	Antinociceptive	Bioactive
[11]	2017	Hyperforin	Hydroxypropyl-β-cyclodextrin	Cutaneous	Wound healing	Bioactive
[44]	2016	Cineole and limonene	Penetration enhancer vehicle	Transdermal	Antifungal	Excipient
[45]	2016	β-citronellene	Invasomes	Transdermal	Hypertension treatment	Excipient
[46]	2016	Squalene	Solid nanoemulsion	Transdermal	Immunization	Excipient
[14]	2016	Paclitaxel	Solid Lipid Nanoparticles	Cutaneous	Antineoplastic	Bioactive
[47]	2016	Madecassoside	Liposomes	Cutaneous	Wound healing	Bioactive
[48]	2016	Asiaticoside	Nanofibers	Cutaneous	Wound healing	Bioactive
[49]	2016	Triterpenoids of *Ganoderma l.*	Nanogel	Cutaneous	Frostbite treatment	Bioactive
[50]	2016	Safranal	Nanoemulsion	Nasal	Cerebral ischemia treatment	Bioactive
[51]	2015	Ursolic acid	Niosomal gel	Transdermal	Antiarthritic	Bioactive
[52]	2015	Farnesol	Polymeric nanoparticles	Oral	Antibiofilm	Bioactive
[53]	2015	Ursolic acid and oleanoic acid	Nanoemulsion	Cutaneous	Anti-inflammatory	Bioactive
[13]	2015	Docetaxel	Nanofibers	Cutaneous	Antineoplastic	Bioactive
[54]	2014	Limonene	Nanoemulsion	Transdermal	NA	Excipient
[55]	2014	Limoneno	PEGilated liposomes	Transdermal	NA	Excipient
[56]	2014	Limonene and 1,8-cineole	Nanoemulsion and solid lipid nanoparticles and nanostructures lipid carriers	Cutaneous	Cutaneous lesions treatment	Excipient
[12]	2014	Paclitaxel	Solid lipid nanoparticles and Nanostructures lipid carriers	Cutaneous	Hyperkeratosis treatment	Bioactive
[57]	2013	Betulin	Nanoemulsion	Cutaneous	Antineoplastic	Bioactive
[58]	2013	Limonene	Nanoemulsion	Transdermal	Analgesic	Excipient
[59]	2013	Astragaloside IV	Solid lipid nanoparticles	Cutaneous	Wound healing	Bioactive
[60]	2013	Limonene	Liposomes	Transdermal	NA	Excipient
[61]	2013	Lupane	Liposomes	Cutaneous	Leishmanicidal	Bioactive
[62]	2013	Hurpezine A	Solid lipid nanoparticles, Nanostructures lipid carriers and Microemulsion	Transdermal	Alzheimer’s treatment	Bioactive
[63]	2012	Tripterine	Nanostructures lipid carriers	Cutaneous	Antineoplastic	Bioactive
[64]	2012	Asiaticoside	Liposomes	Transdermal	Stimulation of collagen synthesis	Bioactive
[65]	2010	Triptolide	Ethosomes	Transdermal	Anti-inflammatory	Bioactive
[66]	2010	Squalene	Nanostructures lipid carriers	Cutaneous	Psoriasis treatment	Excipient
[67]	2009	Cineole	Penetration enhancer vehicle	Transdermal	Alopecia treatment	Excipient
[68,69,70]	2009, 2008	Limonene, citral and cineole	Invasomes	Transdermal	Photosensitization	Excipient
[71]	2005	Triptolide	Solid lipid nanoparticles	Cutaneous	Anti-inflammatory	Bioactive
[72]	2003	Sericoside	derivates of β- and γ-cyclodextrins	Cutaneous	Anti-inflammatory	Bioactive
[73]	2003	Triptolide	Solid lipid nanoparticles	Cutaneous	Anti-inflammatory	Bioactive
[24]	1989	Forskolin	β- and γ-cyclodextrins	Ocular	Treatment of intraocular hypertension	Bioactive

NA: not applicable.

**Table 2:** Publications found in the literature concerning the encapsulation of essential oils, fixed oils, and plant extract containing terpenes in nanostructured systems intended for topical administration.

	Year	Essential Oil/Fixed Oil/Plant Extract	System	Administration Route	Biological Activity	Essential Oil/Fixed Oil/Plant Extract Role in the System
[74]	2018	Lemon EO	Nanoemulsion	Cutaneous	Hyperpigmentation treatment	Excipient
[75]	2018	Clove EO and sweet fennel EO	Nanoemulsion	Cutaneous	Autoimmune dermatoses	Excipient
[76]	2018	Clove EO and sweet fennel EO	Nanoemulsion	Cutaneous	Autoimmune dermatoses	Excipient
[77]	2018	Mentha EO	Nanogel	Vaginal	Antifungal	Bioactive
[78]	2018	*Nigella Sativa* EO	Nanoemulsions	Cutaneous	Anti-inflammatory	Bioactive
[79]	2017	Clove EO and Sweet Fennel EO	Nanoemulsion	Transdermal	Autoimmune dermatoses	Excipient
[80]	2017	*Cymbopogon Flexuous* EO	Nanocapsules	Cutaneous	Antimicrobial	Bioactive
[81]	2017	Rosemary EO	Lipid Nanoparticles	Cutaneous	Skin hydration	Bioactive
[82]	2017	*Eucaliptus globulosus* EO	Nanosized-microemulsion	Cutaneous	Repellent	Bioactive
[83]	2017	*Syzygium aromaticum* EO	Nanoemulsion	Cutaneous	Wound healing and antidermatophytic	Bioactive
[84]	2016	Tea Tree Oil	Nanoemulsion	Cutaneous	Antimicrobial	Bioactive
[85]	2016	*Centella asiatica* extract	Hydroxypropyl-β-cyclodextrin	Cutaneous	Wound healing	Bioactive
[86]	2016	*Lippia sidoides* EO	Nanogel	Oral	Periodontitis treatment	Bioactive
[87]	2015	*Foeniculum vulgare* EO	Nanoemulsion	Transdermal	Antidiabetic	Bioactive
[88]	2015	Lemongrass oil	Nanosponges	Cutaneous	Antifungal	Bioactive
[89]	2015	*Copaifera multijuga EO*	Nanoemulsion	Percutaneous	Anti-inflammatory	Bioactive
[90]	2015	*Santolina insularis* EO	Penetration enhancing vehicle	Percutaneous	Antimicrobial	Bioactive and excipient
[91]	2015	*Melaleuca alternifolia* EO	Nanoemulsion and Nanocapsules	Cutaneous	Wound healing and antidermatotophytic	Bioactive
[92]	2014	Eucalyptus oil	Nanoemulsion	Cutaneous	Antibacterial and Wound healing	Bioactive
[93]	2014	Plai oil	Hydroxypropyl-β-cyclodextrin and Nanofibers	Cutaneous	Anti-inflammatory	Bioactive
[94]	2013	*Stenachaenium megapotamicum* EO	Nanoemulsion	Cutaneous	Antidermatophytic	Bioactive
[95]	2013	*Anethum graveolens* EO	Liposomes	Cutaneous	Antifungal	Bioactive
[96]	2013	*M. alternifolia* EO	Nanoemulsion and nanocapsules	Ungueal	Onychomycosis treatment	Bioactive
[97]	2012	*C. multijuga* EO	Nanoemulsion	Percutaneous	Anti-inflammatory	Bioactive
[98]	2012	Turmeric oil	Nanoemulsion	Cutaneous	Psoriasis treatment	Bioactive
[99]	2009	Citronella oil	Nanoemulsion	Cutaneous	Repellent	Bioactive
[100]	2007	*Artemisia arborescens* EO	Solid Lipid Nanoparticles	Cutaneous	Antiviral	Bioactive
[101]	2004	Viton oil	Liposomes	Cutaneous	Anti-inflammatory	Bioactive

## Supplementary Materials

The following are available online at https://www.mdpi.com/2218-273X/9/4/138/s1, Table S1: Database search lines.
