# Supplementary material for: Essential Oils and Isolated Terpenes in Nanosystems Designed for Topical Administration: A Review
Source: Biomolecules. 2019 Apr 5;9(4):138. doi: 10.3390/biom9040138 (PMC6523335; doi:10.3390/biom9040138)
Supplement: Supplementary file 1 [file biomolecules-09-00138-s001.pdf]

Supplementary Materials

Table S1. Database search lines.

Embase	('essential oil*':ti,ab OR 'volatile oil*':ti,ab OR 'terpene'/exp OR 'monoterpene':ti,ab OR 'monoterpenes':ti,ab OR 'terpene':ti,ab OR 'terpenes':ti,ab OR 'terpenoid'/exp OR 'arylterpenoid':ti,ab OR 'terpenoid':ti,ab OR 'sesquiterpene'/exp OR 'sesquiterpene':ti,ab OR 'sesquiterpenes':ti,ab OR 'sesquiterpenes, eudesmane':ti,ab OR 'sesquiterpenes, germacrane':ti,ab OR 'sesquiterpenes, guaiane':ti,ab OR 'tetracyclic sesquiterpene':ti,ab OR 'triterpene'/exp OR 'triterpene':ti,ab OR 'triterpene series':ti,ab OR 'triterpenes':ti,ab OR 'hemiterpene'/exp OR 'hemiterpene':ti,ab OR 'hemiterpenes':ti,ab OR 'diterpene'/exp OR 'diterpene':ti,ab OR 'diterpenes':ti,ab OR 'sesterterpene'/exp OR 'sesterterpene':ti,ab OR 'sesterterpenes':ti,ab OR 'oilresin':ti,ab OR 'oil resin':ti,ab OR' resinous oil':ti,ab) AND (nano*:ti,ab OR cyclodextrin*:ti,ab OR 'nanocapsule'/exp OR 'nanocapsule':ti,ab OR 'nanocapsules':ti,ab OR 'nanoemulsion'/exp OR 'nano emulsion':ti,ab OR 'nano-emulsion':ti,ab OR 'nanoemulsion':ti,ab OR 'nanogel'/exp OR 'solid lipid nanoparticle'/exp OR 'lipopearls':ti,ab OR 'nanoparticle, solid lipid':ti,ab OR 'solid lipid nanoparticle':ti,ab OR 'solid lipid nanoparticles':ti,ab OR 'self nanoemulsifying drug delivery system'/exp OR 'liposome'/exp OR 'depofoam':ti,ab OR 'liposome':ti,ab OR 'liposomes':ti,ab OR 'phospholipid bilayer vesicle':ti,ab OR 'phospholipid liposome':ti,ab OR 'unilamellar liposomes':ti,ab) AND ('topical drug administration'/exp OR 'administration, topical':ti,ab OR 'drug administration, topical':ti,ab OR 'topic administration':ti,ab OR 'topic application':ti,ab OR 'topic medication':ti,ab OR 'topic therapy':ti,ab OR 'topic treatment':ti,ab OR 'topical administration':ti,ab OR 'topical application':ti,ab OR 'topical drug administration':ti,ab OR 'cutaneous drug administration'/exp OR 'administration, cutaneous':ti,ab OR 'cutaneous administration':ti,ab OR 'cutaneous drug administration':ti,ab OR 'dermal administration':ti,ab OR 'epicutaneous administration':ti,ab OR 'epicutaneous drug administration':ti,ab OR 'mucosal drug administration'/exp OR 'administration, mucosal':ti,ab OR 'mucosal administration':ti,ab OR 'mucosal delivery':ti,ab OR 'mucosal dosage':ti,ab OR 'mucosal dose':ti,ab OR 'mucosal drug administration':ti,ab OR 'mucosal therapy':ti,ab OR 'mucosal treatment':ti,ab OR 'transmucosal administration':ti,ab OR 'transmucosal delivery':ti,ab OR 'transmucosal dosage':ti,ab OR 'transmucosal drug administration':ti,ab)
Scopus	TITLE-ABS-KEY("nano*" OR "cyclodextrin" OR "nanocapsule" OR "nanocapsule" OR "nanocapsules" OR "nanoemulsion" OR "nano emulsion" OR "nano-emulsion" OR "nanogel" OR "solid lipid nanoparticle" OR "lipopearls" OR "nanoparticle, solid lipid" OR "solid lipid nanoparticle" OR "solid lipid nanoparticles" OR "self nanoemulsifying drug delivery system" OR "liposome" OR "depofoam" OR "liposomes" OR "phospholipid bilayer vesicle" OR "phospholipid liposome "OR "unilamellar liposomes") AND TITLE-ABS-KEY("essential oil*" OR "volatile oil*" OR "terpene" OR "monoterpene" OR "monoterpenes" OR "terpenes" OR "terpenoid" OR "arylterpenoid" OR "sesquiterpene" OR "sesquiterpenes" OR "sesquiterpenes,eudesmane" OR "sesquiterpenes, germacrane" OR "sesquiterpenes, guaiane" OR "tetracyclic sesquiterpene" OR "triterpene" OR "triterpene series" OR "triterpenes" OR "hemiterpene" OR "hemiterpenes" OR "diterpene" OR "diterpenes" OR "sesterterpene" OR "sesterterpene" OR "sesterterpenes" OR "oilresin" OR "resinous oil" OR "oil resin") AND TITLE-ABS-KEY("topical drug administration administration, topical" OR "drug administration, topical" OR "topic administration" OR "topic application" OR "topic medication" OR "topic therapy" OR "topic treatment" OR "topical administration" OR "topical application" OR "topical drug administration" OR "cutaneous drug administration" OR "administration, cutaneous" OR "cutaneous administration" OR "cutaneous drug administration" OR "dermal administration" OR "epicutaneous administration" OR "epicutaneous drug administration" OR "mucosal drug administration" OR "administration, mucosal" OR "mucosal administration" OR "mucosal delivery" OR "mucosal dosage" OR "mucosal dose" OR "mucosal drug administration" OR "mucosal injection" OR "mucosal therapy" OR "mucosal treatment" OR "transmucosal administration" OR "transmucosal delivery" OR "transmucosal dosage" OR "transmucosal drug administration")

Pubmed	("nano*" OR "cyclodextrin" OR "nanocapsule" OR "nanocapsule" OR "nanocapsules" OR "nanoemulsion" OR "nano emulsion" OR "nano-emulsion" OR "nanogel" OR "solid lipid nanoparticle" OR "lipopearls" OR "nanoparticle, solid lipid" OR "solid lipid nanoparticle" OR "solid lipid nanoparticles" OR "self nanoemulsifying drug delivery system" OR "liposome" OR "depofoam" OR "liposomes" OR "phospholipid bilayer vesicle" OR "phospholipid liposome "OR "unilamellar liposomes" [TITLE/ABSTRACT]) AND ("essential oil*" OR "volatile oil*" OR "terpene" OR "monoterpene" OR "monoterpenes" OR "terpenes" OR "terpenoid" OR "arylterpenoid" OR "sesquiterpene" OR "sesquiterpenes" OR "sesquiterpenes,eudesmane" OR "sesquiterpenes, germacrane" OR "sesquiterpenes, guaiane" OR "tetracyclic sesquiterpene" OR "triterpene" OR "triterpene series" OR "triterpenes" OR "hemiterpene" OR "hemiterpenes" OR "diterpene" OR "diterpenes" OR "sesterterpene" OR "sesterterpene" OR "sesterterpenes" OR "oilresin" OR "resinous oil" OR "oil resin" [TITLE/ABSTRACT]) AND ("topical drug administration administration, topical" OR "drug administration, topical" OR "topic administration" OR "topic application" OR "topic medication" OR "topic therapy" OR "topic treatment" OR "topical administration" OR "topical application" OR "topical drug administration" OR "cutaneous drug administration" OR "administration, cutaneous" OR "cutaneous administration" OR "cutaneous drug administration" OR "dermal administration" OR "epicutaneous administration" OR "epicutaneous drug administration" OR "mucosal drug administration" OR "administration, mucosal" OR "mucosal administration" OR "mucosal delivery" OR "mucosal dosage" OR "mucosal dose" OR "mucosal drug administration" OR "mucosal injection" OR "mucosal therapy" OR "mucosal treatment" OR "transmucosal administration" OR "transmucosal delivery" OR "transmucosal dosage" OR "transmucosal drug administration" [TITLE/ABSTRACT])
